# A rare case of tender facial nodules as a presenting sign of lymphoplasmacytic lymphoma

**DOI:** 10.1016/j.jdcr.2025.08.028

**Published:** 2025-09-06

**Authors:** Medha Sharma, Caitlyn N. Myrdal, Colin J. Thomas, Jina Chung, Stefan K. Barta, Ellen Kim

**Affiliations:** a Department of Dermatology, Perelman School of Medicine, University of Pennsylvania, Philadelphia, Pennsylvania; b Department of Medicine, University of Pennsylvania, Philadelphia, Pennsylvania; c Abramson Cancer Center, University of Pennsylvania, Philadelphia, Pennsylvania

**Keywords:** lymphoma, lymphoplasmacytic lymphoma, nodule, non-Hodgkin’s, plaque, skin

## Introduction

Lymphoplasmacytic lymphoma (LPL) is a rare subtype of non-Hodgkin’s lymphoma, encompassing <1% of hematologic malignancies in the United States.^1^ Most LPL patients develop a circulating monoclonal IgM, causing the hyperviscosity syndrome of Waldenström macroglobulinemia.^2^ Cutaneous involvement is rare, comprising ∼5% of extramedullary disease.^1^

We report a unique case of an 85-year-old woman without a known history of lymphoma who presented with tender, violaceous nodules and plaques on the nasal tip, ear, and tongue found to be secondary cutaneous LPL.

## Case report

**Fig 1:**
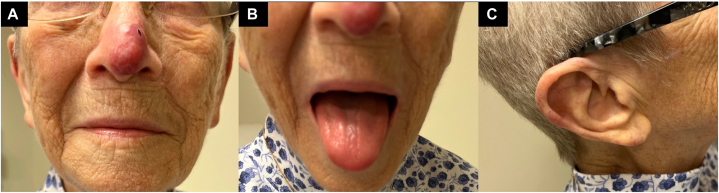
Clinical appearance. **A,** Violaceous nodular plaque on the nasal tip around the prior squamous cell carcinoma excision surgical scar. **B,** Violaceous papules on the tongue. **C,** Erythematous nodules on the right helix and earlobe.

An 85-year-old female with a history of squamous cell carcinoma on the nasal tip excised 2 years ago presented with an erythematous nodular plaque on her nose growing for about a year (Fig 1, *A*). The initial shave biopsy was interpreted at an outside hospital as cutaneous lymphoid hyperplasia, and the lesion was monitored but never resolved. Six months later, the patient developed additional tender, violaceous nodules on the tongue, right helix, and earlobe (Fig 1, *B* and *C*). She did not have B symptoms, organomegaly, lymphadenopathy, hyperviscosity symptoms, or peripheral neuropathy.

**Fig 2:**
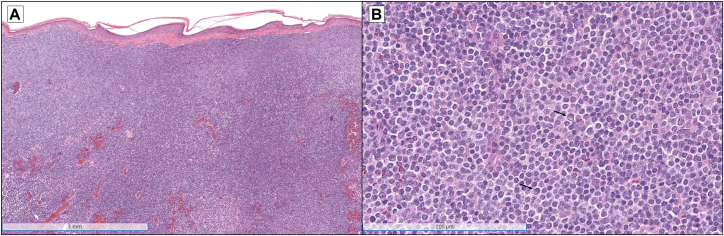
Histopathology of nasal tip skin biopsy. **A,** Low-power view demonstrating an unremarkable epidermis and a Grenz zone, as well as a diffuse sheet-like dermal infiltrate of lymphocytes (hematoxylin and eosin [H&E], 4× magnification). **B,** Higher-power view indicating that the infiltrate is composed of small- to medium-sized lymphocytes admixed with plasma cells (*arrows*) (H&E, 22.4× magnification).

**Fig 3:**
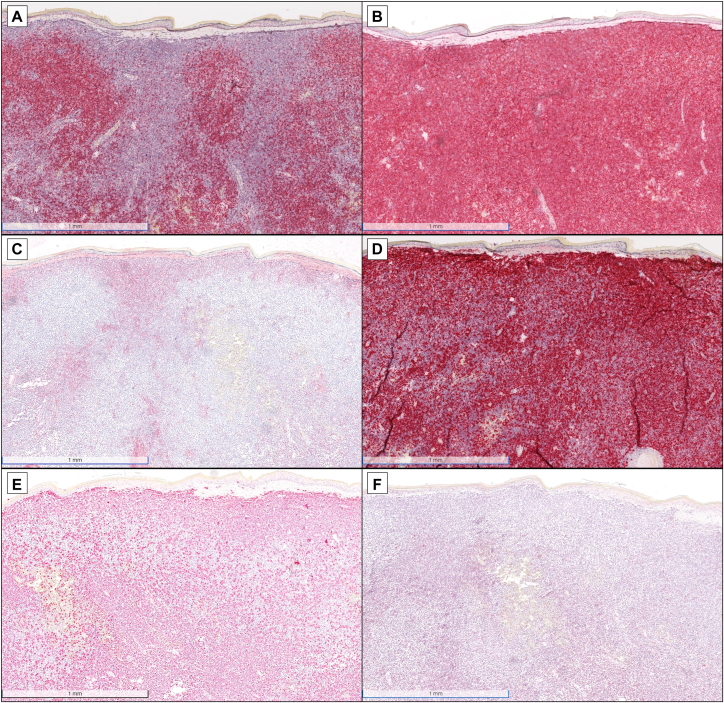
Immunohistochemical and in situ hybridization studies. **A** and **B,** The infiltrate is positive for PAX5 **(A)** and BCL2 **(B)** (4× magnification). **C** and **D,** CD138 **(C)** and MUM-1 **(D)** stains highlighting the plasma cell component, although there is partial loss of CD138 expression within the plasma cells (4× magnification). **E** and **F,** Kappa **(E)** and lambda **(F)** in situ hybridization studies exhibiting kappa light chain restriction (4× magnification).

Complete blood count and differential were normal without cytopenias. A biopsy of the nasal tip demonstrated an unremarkable epidermis and a Grenz zone, a diffuse sheet-like dermal infiltrate of mature to atypical plasma cells, and numerous small-to-medium-sized lymphocytes (Fig 2). Immunohistochemical stains revealed that the infiltrate was positive for PAX5 and BCL2 (Fig 3, *A* and *B*). CD138 and MUM-1 stains highlighted the plasma cell component, although there was partial loss of CD138 expression (Fig 3, *C* and *D*). Kappa and lambda in situ hybridization studies demonstrated kappa light chain restriction (Fig 3, *E* and *F*). Immunoglobulin heavy chain gene rearrangement studies by polymerase chain reaction were performed on the skin biopsy specimen, which was positive for a clonal rearrangement; *MYD88* mutation analysis revealed an L265P mutation.

Serum protein electrophoresis demonstrated a 0.5 g/dL M-spike identified as IgM kappa on immunofixation. Immunofixation also exhibited a faint IgG kappa band in the far gamma region. Quantitative immunoglobulin assay revealed elevated IgM at 1386 mg/dL. Kappa quantitative free light chains were elevated at 184.89 mg/L, and the kappa/lambda ratio was elevated at 8.54. Peripheral blood flow cytometry exhibited a kappa-restricted, clonal B-cell population comprising 45.9% of lymphocytes, with a CD5− CD10− CD20+ immunophenotype similar to what was observed on the skin biopsy. These findings were consistent with secondary cutaneous involvement from LPL originating in the lymph nodes or bone marrow.

Staging positron emission tomography/computed tomography scan revealed uptake in the nasal tip, a hypermetabolic lung nodule of uncertain etiology, possibly representing LPL versus a new primary lung malignancy, and low-level uptake in the precarinal and subcarinal lymph nodes. Given patient preference, age, and comorbidities, the lung nodule was monitored, bone marrow biopsy deferred, and 4 weekly rituximab doses initiated with a Bruton’s tyrosine kinase inhibitor on reserve. Rituximab resulted in improvement of her plaques and nodules, lymphadenopathy, and peripheral blood IgM quantity.

## Discussion

Secondary cutaneous manifestations of LPL typically result from tissue deposition of IgM paraproteins and can present as cutaneous amyloidosis, purpura, or ulcers.^2^ Direct dermal infiltration by neoplastic lymphoid cells, as seen in our patient, is rarely the presenting symptom.^3^ Morphologies are variable, with prior reports describing erythematous to violaceous papules, plaques, nodules, tumors, and ulcers, usually on the trunk and extremities.^1^^,^^3^ The ears and face are uncommon sites of involvement, and to our knowledge, there have been no prior reports of cutaneous LPL on the mucosa.^3^ Additionally, our case is unique, as cutaneous lesions typically present years after a known diagnosis of Waldenström macroglobulinemia.^3^

The primary competing diagnosis was primary or secondary cutaneous marginal zone lymphoma (MZL), which often mimics LPL on histopathology. The strongest clue favoring an LPL diagnosis in our patient was a L265P mutation in the *MYD88* gene. Though not entirely specific, the mutation has been identified in 95% to 97% of patients with LPL compared to only 7% of patients with MZL, with a higher prevalence in MZL patients that are not class-switched to IgM+.^4^^,^^5^ This activating point mutation enhances B-cell survival.^6^

In 70% of patients, the bone marrow and peripheral blood are involved at the time of LPL diagnosis; thus, bone marrow biopsies are recommended.^2^ A diagnosis can be made if 10% or more of the biopsy sample exhibits neoplastic infiltration by small lymphocytes, plasmacytoid lymphocytes, and plasma cells admixed with immunoblasts alongside characteristic mast cell hyperplasia.^7^ The infiltrate is often surface IgM+, CD5−/+, CD10−, CD19+, and CD20+.^8^ Our patient deferred bone marrow biopsy, but the peripheral blood immunotype was consistent with LPL. Cutaneous deposition of IgM paraproteins may correlate with higher disease severity compared to cases with dermal infiltration of neoplastic lymphoid cells, though too few cases have been reported to draw strong prognostic conclusions.^3^

Patients with cutaneous LPL have demonstrated complete remission with 6 weeks of radiation^9^ or rituximab and bendamustine^4^ or cyclophosphamide and dexamethasone.^6^ Most lesions recurred within 5 years but were successfully re-treated with ibrutinib,^2^ chemotherapy, radiation, or excision.^9^ Given our patient’s multifocal cutaneous disease, rituximab was initiated. Interestingly, some previous case reports have observed progression following rituximab monotherapy, but our patient experienced clinical improvement.^10^

In conclusion, we present a unique case of secondary cutaneous LPL presenting as tender, violaceous nodules and plaques on the ear, tongue, and nasal tip, treated successfully with rituximab. Though secondary cutaneous LPL is rare, especially as a presenting symptom, it should be considered in the differential of tender, violaceous skin plaques, particularly if the biopsy reveals a mature B-cell lymphoma with plasmacytic differentiation.

## Conflicts of interest

None disclosed.
